# Recombinant Expression Screening of *P. aeruginosa *Bacterial Inner Membrane Proteins

**DOI:** 10.1186/1472-6750-10-83

**Published:** 2010-11-29

**Authors:** Vidya Madhavan, Forum Bhatt, Constance J Jeffery

**Affiliations:** 1 Laboratory for Molecular Biology, Department of Biological Sciences, University of Illinois at Chicago, Chicago, IL 60607, USA

## Abstract

**Background:**

Transmembrane proteins (TM proteins) make up 25% of all proteins and play key roles in many diseases and normal physiological processes. However, much less is known about their structures and molecular mechanisms than for soluble proteins. Problems in expression, solubilization, purification, and crystallization cause bottlenecks in the characterization of TM proteins. This project addressed the need for improved methods for obtaining sufficient amounts of TM proteins for determining their structures and molecular mechanisms.

**Results:**

Plasmid clones were obtained that encode eighty-seven transmembrane proteins with varying physical characteristics, for example, the number of predicted transmembrane helices, molecular weight, and grand average hydrophobicity (GRAVY). All the target proteins were from *P. aeruginosa*, a gram negative bacterial opportunistic pathogen that causes serious lung infections in people with cystic fibrosis. The relative expression levels of the transmembrane proteins were measured under several culture growth conditions. The use of *E. coli *strains, a T7 promoter, and a 6-histidine C-terminal affinity tag resulted in the expression of 61 out of 87 test proteins (70%). In this study, proteins with a higher grand average hydrophobicity and more transmembrane helices were expressed less well than less hydrophobic proteins with fewer transmembrane helices.

**Conclusions:**

In this study, factors related to overall hydrophobicity and the number of predicted transmembrane helices correlated with the relative expression levels of the target proteins. Identifying physical characteristics that correlate with protein expression might aid in selecting the "low hanging fruit", or proteins that can be expressed to sufficient levels using an *E. coli *expression system. The use of other expression strategies or host species might be needed for sufficient levels of expression of transmembrane proteins with other physical characteristics. Surveys like this one could aid in overcoming the technical bottlenecks in working with TM proteins and could potentially aid in increasing the rate of structure determination.

## Background

Ion transport, cell-cell communication, vesicle transport, maintenance of cellular structure, drug resistance, host-pathogen interactions and many other vital cellular activities involve proteins that are embedded in the cell membrane. Transmembrane [TM] proteins make up over 25% of an organism's proteins [1-3] and are the targets for the majority of pharmaceuticals in use today [4]. The improper folding or activity of TM proteins lead to important genetic diseases, including cystic fibrosis and diabetes. The variety of roles of transmembrane proteins in physiological functions important to both medicine and basic science make the determination of transmembrane protein structures and molecular mechanisms an important goal, but, in spite of their vast importance, there are far fewer structures and molecular mechanisms known for TM proteins than for soluble proteins. While there are almost two hundred structures of alpha-helical membrane proteins in the PDB, problems in transmembrane protein expression, solubilization, and structure determination have led many researchers instead to focus on soluble proteins. Currently, the majority of structural genomic projects focus on the soluble proteins, the "low-hanging fruit", due to their relative ease of expression, purification, and crystallization.

**Figure 1:**
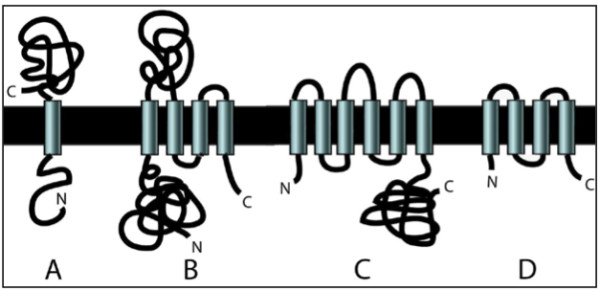
**Schematic diagram of TM protein topology**. The number of TM helices, the lengths of the N- and C-terminal domains, and the sizes of the interhelical loops or domains vary. Some TM proteins are located predominantly within the membrane (D), but others have more extensive N- or C-termini and/or cytoplasmic or periplasmic loops (A, B, C) that can form independently folding globular domains.

One of the key bottlenecks in studying transmembrane proteins has been expression of sufficient amounts of protein for biochemical and structural characterization. For many soluble proteins and some transmembrane proteins, recombinant expression in *E. coli *provides a sufficient amount of protein for many types of studies and has the advantages of ease of use, low cost, and ability to be scaled up. Some transmembrane proteins can be expressed in *E. coli *at sufficient levels for biochemical studies, but it is not clear which ones. Transmembrane proteins vary in the number of transmembrane helices (Figure 1), size, overall hydrophobicity, size of domains outside the membrane, and other physical features that might play a role in determining if a transmembrane protein will be expressed at high levels in *E. coli*. Identifying those physical features that affect recombinant expression levels could facilitate the identification of proteins that could be expressed at sufficient levels for biochemical and structural studies.

We have performed a systematic study of recombinant expression of transmembrane proteins in *E. coli*. We have selected a medically important gram-negative bacterium, *Pseudomonas aeruginosa*, as the source of the proteins. Using proteins from a bacterium avoids many of the potential complications that might affect expression of human or other mammalian proteins such as lack of glycosylation or other post-translational modifications. *Pseudomonas aeruginosa *is an opportunistic bacterial pathogen that infects burn patients, immunocompromised patients, and cystic fibrosis patients. Cystic fibrosis is the most common lethal genetic disease in North America. Chronic lung infections by *P. aeruginosa *and the inflammatory host immune response are major causes of the progressive lung damage and low life expectancy of CF patients. *P. aeruginosa *is also a common cause of nosocomially-acquired infections due to its intrinsic resistance to many drugs. Better methods to eradicate *P. aeruginosa *infections are needed to help decrease lung damage in CF patients and increase the life span of CF, burn, immunocompromised, and other patients. Of particular interest are the numerous transmembrane proteins involved in antibiotic resistance and efflux, pathogen-host interactions, cell-cell signaling, quorum sensing, and other steps in infection and virulence. In addition to specific proteins involved in these processes, *P. aeruginosa *provides many other important transmembrane protein targets for studying activities like transport and signaling, as well as for studying transmembrane protein structure and folding.

We tested the recombinant expression of eighty-seven transmembrane proteins from *P. aeruginosa *with a variety of physical features and functions to determine if those factors play a role in relative expression level. We also evaluated several culture growth conditions in order to assess which provided superior protein expression.

## Results and Discussion

### Selection of Proteins

The vast majority of transmembrane proteins are predicted to have membrane-embedded regions composed of alpha-helices [reviewed in [5-7]]. Exceptions include proteins in the outer membrane of gram-negative bacteria, mitochondria and chloroplasts and some toxins. For this study, we are focusing on transmembrane proteins for which the membrane-embedded domain is predicted to be made up of transmembrane helices. Hydropathy analysis indicates that *Pseudomonas aeruginosa *encodes 1334 proteins that are predicted to have at least 1 transmembrane helix (out of 5568 predicted genes in the genome), however, a single predicted transmembrane helix might be a cleaved signal sequence, so only those proteins with two or more predicted TM regions were included, of which there are 930. Target proteins were selected based on several physical features and to include proteins with a variety of functions. Most proteins annotated as a "subunit" were not selected for the study because attempts to express a subunit of a larger multimer might not result in correctly folded and stable protein. Our selection strategy provides target proteins with a variety of physical characteristics and functions for the expression tests. It should be noted that correlation of individual characteristics with protein expression levels might not be simple and clear-cut. Several factors are likely to contribute to the results, for example the number of TM helices in combination with the sizes interhelical loops. Because of this, a large number of protein targets were selected and general trends were studied.

### Cloning Steps

Invitrogen's Gateway system was chosen for cloning and expressing many proteins in parallel, and has been used by several groups for high-throughput protein expression [8,9]. Benefits for potential future projects using these plasmids include: [1] The Entry Clone enables quick transfer the cloned gene of interest into additional vectors, which can enable expression in different organisms or can include features like different affinity tags. (2) Bacterial cultures can be scaled up for selected proteins, for future work on purification and biochemical or structural characterization. (3) The 6-histidine (6-His) affinity tag added for Western blot analysis can also be used for purification of selected target proteins using nickel chelating columns. (4) The plasmids can be used for expression of selected target proteins in auxotroph strains for incorporation of selenomethionine or selenocysteine, respectively, to aid in structural determination by MAD phasing [10]. We obtained through cloning and from the Harvard Proteomics lab and Open Biosystems a total of 87 plasmids for our study. The proteins studied are listed in Additional file 1.

### Expression

Protein expression was tested in two strains and up to four different temperatures (Table 1). *E. coli *BL21-AI is a commonly used expression strain that is a derivative of BL21(DE3). CD43(DE3) is also a derivative of BL21(DE3) and was reported to overproduce TM proteins with less toxicity [11]. The expression experiments were repeated at multiple temperatures (37°C, 30°C, 20°C, and 12°C for BL21-AI, and 37°C and 30°C for CD43(DE3)). Expression at lower temperatures is expected to lower the translation rate, which might slow down demands on the translocon and also might provide the target proteins with an increased chance of folding correctly. Overall, we observed expression of 61 out of 87 target proteins, or 70%. It should be noted that, although we refer to our studies as "expression studies", a number of factors, including rate of expression and stability of the protein, contribute to the amount of protein observed: Some proteins might not appear on the Western blots because they are (a) unstable or (b) normally found in the cell as part of a complex with other proteins (or a cofactor) and aren't properly folded or stable without concurrent expression of other members of the complex or (c) normally interact with cytosolic or peripheral proteins that are limited in these overexpressing conditions, or (d) are toxic to the cell. Because we do not have all this information for every target protein, we were unable to correct for these kinds of nonexpressed proteins, but the large number of target proteins in the study helped us look for general trends.

**Table 1:** Expression Results Based on Strain and Temperature.

Strain	Temperature	Number of Proteins Tested	Positive Expression	Percent of Proteins Expressed
BL21-AI	14 °C	85	32	38%
BL21-AI	20 °C	85	49	58%
BL21-AI	30 °C	85	48	56%
BL21-AI	37 °C	85	22	26%
C43(DE3)	30 °C	87	27	31%
C43(DE3)	37 °C	87	42	48%

It is interesting to note that of the twenty proteins that were annotated as "hypothetical" or "unknown", nine were expressed and eleven were not (45% expressed). This number is lower than the number that were expressed of the proteins with a known function or with a predicted or "probable" function based on sequence similarity (78% expressed). One possibility is that proteins that are difficult to express using recombinant DNA methods are less likely to be characterized, and it is possible that their homologues from other species might also be difficult to express using recombinant DNA methods.

**Figure 2:**
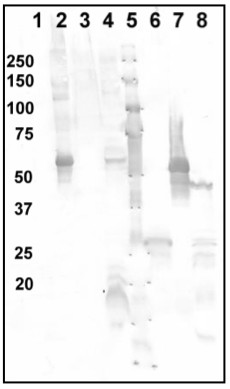
**Example of the Western blots used to estimate relative expression levels**. Proteins were expressed in strain BL21-AI at 37°C. Whole cell extracts were subjected to SDS-PAGE and transferred to membranes. The blots were probed with anti-6-His antibodies. Overall, approximately 65% of the proteins were expressed in at least one condition, and the sizes of most of the expressed proteins were found to correspond approximately with their predicted sizes. Lane 1 PA2628 (MW = 32 kDa), Lane 2 Control 6-His tagged protein (MW = 60 kDa), Lane 3 PA3773 (MW = 39 kDa), Lane 4 PA5291 (MW = 73 kDa), Lane 5 Molecular weight markers with weights given in kDA, Lane 6 PA4083 (MW = 26 kDa), Lane 7 PA4417 (MW = 51 kDa), Lane 8 PA5199 (MW = 49 kDa).

Expression of the target proteins was monitored by Western blotting using anti-6-His tag antibodies (Figure 2). Other labs noted difficulties in the transfer of larger, more hydrophobic membrane proteins to the blot [12], but we did not observe this difficulty. The majority of lanes in the Western blots had at least one additional band in addition to the band running at the predicted molecular weight, although these other bands were usually lighter than the band at the predicted molecular weight. Using Western blots with anti-6-His tag antibodies allowed us to estimate the expression levels of the full-length proteins, ignore these background or nonspecific bands, and at the same time to check for protein size and potential degradation products. Other methods, such as dot-blots, do not allow for this assessment. It has previously been noted that membrane transport proteins often migrate on SDS-PAGE gels at only 65-75% of their true molecular weight, possibly due to their hydrophobicity, binding of relatively large amounts of SDS, or their retention of some alpha-helical secondary structure enabling them to move more quickly through the gel [13]. For most of the *P. aeruginosa *proteins in our study, a band was observed at approximately the expected molecular weight. In some cases, additional bands corresponding to lower molecular weight species were detected, suggesting that some proteolysis of the TM protein may have occurred. For a few proteins, a smear was observed running at higher molecular weight than the main band for the protein, possibly due to nonspecific aggregation. Thirty-eight of the 58 expressed proteins were expressed with no smearing at any temperature in any strain, and 10 expressed proteins had smears in half of the conditions tested. Because the majority of the proteins had smears in more than one condition, it is likely that it is a characteristic of the protein, not the preparation.

Proteins that were expressed tended to be expressed at more than one temperature or in more than one strain. Of the 61 proteins that were expressed in at least one condition in this study, twelve were expressed in all six conditions and twelve were expressed in only one condition. The highest number of proteins was expressed in BL21-AI at 20°C, which included three proteins that were not expressed at any other condition. One protein that was not expressed in any other condition was expressed in BL21-AI cells at 14°C and another six were expressed only in BL21-AI cells at 30°C. More bands that appeared to be degradation products were observed for both expression strains at higher temperatures. With strain BL21-AI, degradation products were observed for 6 proteins at 14°C, 8 at 20°C, 19 at 30°C and 17 at 37°C. With strain C43 degradation products were observed for 4 proteins at 30°C and 11 at 37°C. In some cases the level of expression of a single protein in different conditions varied from not expressed to the highest level of expression.

As the majority of the proteins that were expressed in only one condition were expressed in BL21-AI at 30°C, it would appear to be a good culture growth condition to try first. However, since this condition also resulted in the most proteins with degradation projects, it would also be good to test strain C43(DE3), in which fewer proteins were observed to have additional, lower molecular weight bands that are likely to be degradation products.

### Correlations with Physical Features

Several physical characteristics of the proteins were predicted by sequence analysis and compared to the expression levels. Those factors that appeared to correlate with expression were the number of TM helices (TMH), the percentage of amino acids in TMH, and the grand average of hydropathicity.

#### Number of transmembrane helices

**Figure 3:**
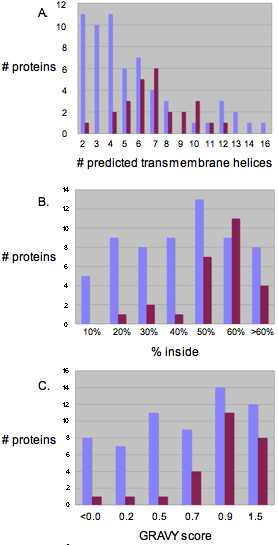
**Summary of correlation of expression results with physical features of the transmembrane proteins**. For each category of each characteristic, the numbers of proteins expressed (grey bars) and not expressed (black bars) are indicated. A. Number of transmembrane helices. Transmembrane proteins with fewer TMH tended to be expressed more often than those with a greater number of TMH. Those proteins that were not expressed had on average more than 7 TMH, while those that were expressed had on average fewer than 6 TMH (Chi-square test p value = 0.005). B. Percentage of amino acids in transmembrane helices versus in periplasmic or cytoplasmic loops and domains. Proteins that were expressed tended to have fewer of their amino acids in the TMHs than those proteins that were not expressed (Chi-square test for trend p value = 0.005). C. Grand average of hydropathicity (GRAVY). Proteins that were not expressed had a higher average GRAVY score (Chi-square test for trend p value 0.008).

Transmembrane proteins with fewer TMH tended to be expressed more often than those with a greater number (Figure 3A). Those proteins that were not expressed had on average more than 7 TMH, while those that were expressed had on average fewer than 6 TMH (Chi-square test p value = 0.005). However, some of the higher molecular weight proteins were expressed, in fact, proteins with has many as twelve TMH were observed to have good levels of expression.

#### Percentage of amino acids in transmembrane helices

Expression levels were also compared for proteins with different percentages of amino acids inside the transmembrane helices versus in periplasmic or cytoplasmic loops and domains. Proteins that were not expressed tended to have more of their amino acids in the TMHs than those proteins that were expressed (Figure 3B, Chi-square test for trend p value = 0.005).

#### Grand average of hydropathicity (GRAVY)

The GRAVY score [14] is a global descriptor of hydropathy. It is the sum of hydrophobicity values for each of the residues in the protein, normalized according to the protein length. More hydrophobic proteins have positive GRAVY values, more hydrophilic proteins have negative values. Proteins that were not expressed had a higher average GRAVY score (Figure 3C, Chi-square test for trend p value 0.008). This difference in average GRAVY scores seems to stem from the lack of low GRAVY scores in transmembrane proteins that were not expressed. There were thirty-five expressed transmembrane proteins with GRAVY scores below 0.70, while only seven transmembrane proteins that were not expressed had GRAVY scores below 0.70. It would seem that a lower GRAVY score is associated with a higher chance of successfully expressing a transmembrane protein or with stability in the membrane.

The correlation of expression with the number of transmembrane helices, the percentage of amino acids in transmembrane helices, and the GRAVY score does not appear to be due to the method used to determine expression level, PAGE gels and Western blots, because in each of these cases, some of the proteins with more TMH, more amino acids in the transmembrane helices, and high GRAVY scores were found to be highly expressed, which indicates that the method used to estimate expression levels did not bias the results due to problems in electrophoresis or transfer to the blotting membrane.

Among those traits which did not correlate with a statistically significant difference in transmembrane expression were the length of the N-terminus before the first transmembrane helix, length of the C-terminus after the last transmembrane helix, molecular weight of the protein, the number of rare codons (AUA, AGG, AGA, CUA, CCC, CGG, and GGA), the number of amino acids in cytoplasmic or periplasmic loops, or in the first loop (between TM1 and TM2), or in the largest loop, the presence or absence of a signal peptide, or the length, hydrophobicity or amphiphilicty of a signal peptide.

In the study described above, the relative expression levels of the transmembrane proteins were measured under several culture growth conditions. The use of *E. coli *strains, a T7 promoter, and a 6-His C-terminal affinity tag resulted in the expression of 61 out of 87 test proteins (70%). During the time that the above experiments were being performed, the results of several other surveys of recombinant TM protein expression in *E. coli*, *Lactococcus lactis*, or *Saccharomyces cerevisiae *were published. In contrast to our work with proteins from *P. aeruginosa*, most of the studies tested expression of TM proteins from *E. coli *or from a thermophile. A few studies included TM transporters or other proteins from pathogens. One study tested the recombinant expression of yeast TM proteins in a yeast host strain.

Nordlund and coworkers studied the recombinant expression of 49 *E. coli *transmembrane proteins with eight or more transmembrane helices in *E. coli *[15]. They used several N-terminal affinity tags and a C-terminal 6-His tag. They also used multiple *E. coli *expression hosts: BL21, C43, and C41. Overall, they reported 71% of the target proteins were expressed, and they found that protein expression worked best with low temperatures and low IPTG concentrations. Hendersons and coworkers studied the recombinant expression of transporters and receptors from several pathogenic bacterial species expressed with a C-terminal 6-His tag [16]. Optimization of expression conditions resulted in expression of 34 out of the 40 proteins studied. The Michel group studied the recombinant expression of 37 target proteins with at least 3 and usually 8 or more TMH, including secondary transporters from *Salmonella *and two hyperthermophiles [12]. They compared a variety of expression conditions including three different vectors in *E. coli *and testing *Lactococcus lactis *as a second expression host. Overall they observed that 78% of the proteins were expressed in the *E. coli *host. When they tested expression of a subset of the target protein in *Lactococcus lactis*, only 40% of the subset were expressed.

Several additional groups performed expression studies and looked for correlation between protein characteristics and expression levels. As was observed for the *P. aeruginosa *TM proteins, three groups observed that recombinant expression of TM proteins correlated with protein characteristics related to overall hydrophobicity or the number of predicted transmembrane helices. Dobrovsky and coworkers tested the expression of 280 proteins from *E. coli *and 77 from a thermophile, *Thermotoga maritima *[17]. Overall they found that there was no advantage to using T. *maritima *proteins because a slightly higher number of proteins from *E. coli *were expressed. As in our study, they observed that the majority of successfully expressed and purified TM proteins had six or fewer transmembrane domains: 43% (54 out of 126) with 6 or fewer TMH were expressed, but only 18% (28 out of 154) were expressed that had more than six TMH. The Cross lab studied the expression of 99 transmembrane proteins from *Mycobacterium tuberculosis *in *E. coli *[18] and observed that 70% were expressed. They observed expression of 64% of the proteins with three or more TMH, but that number increased to 78% of the proteins with only one or two TMH. The Dumont lab studied the expression of 1092 eukaryotic membrane proteins, from *S. cerevisiae*, in a *S. cerevisiae *host strain [19]. In that study about 50% of the proteins that contain five or fewer TMH were expressed at the highest levels, but fewer than 20% of the proteins that contain seven or more THM were expressed at high levels. They also compared the expression of proteins with respect to the amount of each protein predicted to be in the membrane. Over 50% of the proteins predicted to have 20% or less of their amino acids in TMH were highly expressed, but only 30% of the proteins predicted to have more than 20% of their amino acids in the TMH were highly expressed.

One group reported a lack of correlation between the number of predicted TM helices and expression of TM proteins. DeGier and coworkers [20] developed a novel method for determining the level of expression of a membrane protein in *E. coli*. They selected only proteins that are predicted to have the C-terminus located in the cytoplasm and expressed each protein as a C-terminal GFP fusion protein. The resulting cytoplasmic GFP tag was used to measure the amount of expression. This method was later used as part of a larger scale study of transmembrane protein topology [21]. In the latter study, 397 TM proteins that were predicted to have a cytoplasmic C-terminus were tested for expression as a C-terminal GFP fusion protein in *E. coli*. In this study, the authors note that they did not find a correlation between protein expression and sequence characteristics including codon usage, protein size, hydrophobicity, and number of transmembrane helixes. It is not clear why the correlations seen by other labs were not observed in this study, although differences in the methods include the use of only *E. coli *target proteins, the target proteins only included proteins with C-terminus in the cytoplasm, the proteins were expressed as GFP fusion protein, and GFP fluorescence was used to measure expression levels.

It is possible that the tendency for proteins with fewer TMH to be more likely to be expressed might be due to the method of biosynthesis of TM proteins. Perhaps proteins with a larger number of transmembrane helices are more likely to require more time for correct membrane insertion folding. This greater time requirement could lead to less protein produced. Also, proteins with many TMH may tax the membrane insertion system, leading to incorrect insertion or a higher propensity for misfolding. If the translocation machinery required for insertion of the protein into the lipid bilayer during synthesis is limiting, perhaps fewer TM helices passing through the machinery would allow more copies of the protein to be made. If this model is correct, it could result in a trend: better overexpression of proteins with fewer TM helices than with more TM helices. Of course, additional factors might help regulate expression levels for individual proteins or specific types (kinases, channels, etc.) by degradation, for example.

## Conclusions

The variety of roles of transmembrane proteins in physiological functions important to both medicine and basic science make the determination of transmembrane protein structures and mechanisms an important goal. While quite feasible, as demonstrated by the presence of almost two hundred structures of alpha-helical membrane proteins in the PDB, problems in transmembrane protein expression, solubilization, purification, and structure determination have led many researchers instead to focus on soluble proteins.

Surveys like this one could aid in overcoming the technical bottlenecks in working with TM proteins and could potentially aid in increasing the rate of structure determination. They can help identify those factors that contribute to the technical problems so that they can be specifically addressed in the future with novel methods. In the mean time, identifying physical characteristics that correlate with protein expression might aid in selecting the "low hanging fruit of membrane proteins", or proteins that can be expressed to sufficient levels using an *E. coli *expression system. For example, in this study, the target proteins were from *P. aeruginosa*, a gram negative bacterial opportunistic pathogen that causes serious lung infections in people with cystic fibrosis, and several TM proteins involved in infection were found to be expressed at sufficient levels for further study. The use of other expression strategies or host species might be needed for increased levels of expression of transmembrane proteins with other physical characteristics.

Specifically, the above study of *P. aeruginosa *TM proteins and other reports indicated that a large percentage of TM proteins can be expressed in *E. coli*, although several labs observed that lower levels of expression were observed for proteins with larger numbers of TMH. It should also be noted that the expression levels for the TM proteins in all of these studies, where noted, were still far below those of soluble proteins, and varying vector, host strain or species, temperature, and other expression conditions helped but did not vastly improve expression. The next step in producing large amounts of many transmembrane proteins for biochemical and structural studies might require development of a novel expression host that is specially tailored for TM protein expression [22]. Our large collection of plasmid vectors encoding TM proteins varying in molecular weight, hydrophobicity, function, and other characteristics could be used for these future studies.

Overcoming the technical problems in working with TM proteins could potentially have a huge payoff by increasing the rate of determining structures and mechanisms of TM proteins, which make up 25% of all proteins and are key to many physiological processes in both health and disease.

## Methods

### Materials

Genomic DNA from *Pseudomonas aeruginosa *was obtained from the ATCC (Manassas, VA). PCR primers were synthesized by Integrated DNA Technology (Coralville, Iowa). Pfu DNA polymerase, PCR reaction kits, and PCR reaction buffers were purchased from Stratagene (La Jolla, CA). dNTP mixes and p^ENTR^/SD/D-TOPO cloning kits were purchased from Invitrogen Life Technologies (Carlsbad, CA). PCR product purification kits and DNA plasmid miniprep kits were purchased from Qiagen (Valencia, California). Restriction enzymes were purchased from MBI Fermentas (Hanover, MD).

### Sequence Analysis and Selection of Target Proteins

The initial selection of target transmembrane proteins was performed by identifying proteins predicted to have at least two transmembrane helices as annotated in the PEDANT database [23,24] and also predicted by TMPRED [25]. In addition, target proteins were selected so as to include in the collection proteins that vary in physical features such as molecular weight, number of predicted transmembrane helices, overall hydropathy, etc. and predicted functions. The names and types of protein (enzyme, transporter, etc.) were derived from the annotation of all of the *P. aeruginosa *ORFs performed by the *P. aeruginosa *Community Annotation Project [26,27]. TMPRED [25] was utilized to predict the number and locations of transmembrane helices. Calculations of the GRAVY hydropathy index were performed using the ProtParam tool of the Expasy Proteomics server [28,29]. Calculations concerning signal sequences were calculated by Signal P [30,31] and Phobius [32].

### Construction of TM Protein Gene Collection

The *Gateway™ *(Invitrogen) cloning technology system was used for construction of the expression plasmids encoding the transmembrane proteins of interest. The genes encoding the target proteins were amplified by PCR from genomic *P. aeruginosa *DNA (ATCC number 47085 D *Pseudomonas aeruginosa *PA01-LAC). PCR primers were designed with the computer program Clone Manager (Scientific and Educational Software, Cary, NC) using the following criteria: Each forward primer contained a CACC sequence at the 5' end. The CACC sequences base pairs with the overhang sequence, GTGG, in the p^ENTR^/SD/D-TOPO vector (Invitrogen). Each reverse primer was designed to remove the native stop codon in the gene of interest for addition of a C-terminal tag to the protein. Primer pairs were designed so that they had similar melting temperatures (between 50° C and 80° C) and were complimentary to the template. The primers were synthesized by Integrated DNA Technologies (Coralville, IA). PCR products that contained non-specific (unexpected) products were purified using a gel purification kit (Qiagen) and re-analyzed by agarose gel electrophoresis.

The TOPO^® ^Cloning Reaction Kit (Invitrogen) was used to insert each gene into the p^ENTR^/SD/D-TOPO vector to construct the "Entry Clone" plasmids. Cloning reaction products were used to transform chemically competent One Shot^® ^TOP 10 *E. coli *cells (Invitrogen), and miniprep DNA from transformants were digested with restriction enzymes (MBI Fermentas) and analyzed by agarose gel electrophoresis to verify the presence of the gene of interest. The high GC content of the *P. aeruginosa *DNA caused difficulty in the cloning steps, making it difficult to obtain Entry clones of some of the longer proteins. In order to increase the number of clones for the expression studies, additional Entry Clone plasmids were obtained from the Harvard Proteomics Institute or from Open Biosystems (Huntsville, AL).

An Entry Clone plasmid with the correct insert for each gene was then used to transfer the gene into pET-DEST42 to construct the "Destination Clone" plasmids. This vector features the T7 lac promoter for IPTG-inducible expression of the target gene and adds a C-terminal 6-His tag to the target protein. Each LR recombination reaction between an Entry clone and the pET-DEST42 Destination Vector was performed using Clonase Reaction buffer (Invitrogen). The reactions were used for transforming library efficiency DH5α cells (Invitrogen). Restriction digests and gel electrophoresis of DNA minipreps (Qiagen) were used to check for the correct formation of PCR products and Entry and Destination clones. To check for the absence of mutations in the cloned gene sequences in the Destination Clone plasmids, DNA sequencing was performed at the University of Chicago DNA facility.

### Expression Studies

Destination Vectors were used to transform *E. coli *strain Bl21-AI (F-ompT hsdSB (rB-mB-) gal dcm araB::T7RNAP-tetA) (Invitrogen) (with lon and ompT protease deficiencies (Invitrogen), which has the T7 RNA polymerase under the control of the araBAD promoter for inducible expression, and *E. coli *strain C43(DE3) (which was derived fromBL21(DE3) [*E. coli *F-ompT hsdSB (rB-mB-) gal dcm (DE3)]) [11]. For each expression experiment, overnight cultures (LB with 100 μg/ml of ampicillin) were inoculated from fresh transformants or frozen permanents and grown at 37°C with shaking and used to inoculate fresh LB medium containing 100 μg/ml ampicillin (1:20 dilution of the initial culture) in the morning. The cultures were then grown at 37°C until they reached mid-log phase (OD_600 _= 0.4 - 0.6). Expression of the target TM protein was induced with the addition of L-arabinose to a final concentration of 0.2% w/v and IPTG to a final concentration of 1 M. Incubation was continued for five hours after induction for 30°C or 37°C cultures, or twenty-four hours for 14°C or 20°C cultures. (Controls included cultures not treated with arabinose and IPTG (uninduced), and those transformed with vector alone.) 1 ml samples of the cultures were taken and prepared for use in Western blots. The samples were centrifuged, resuspended in 1× Laemmli sample buffer containing 5% v/v β-mercaptoethanol, vortexed thoroughly, boiled in a heat block at 90°C for 5 minutes, and subjected to SDS-PAGE. The presence of the target protein was detected by rabbit anti-6-His tag primary antibodies (cat# RDI-HISTAG1abr, Research Diagnostics) with goat anti-rabbit-AP secondary antibody (GAR-AP control 91126 from BioRad). The Western blots were scored visually, and bands migrating near the predicted molecular weight were scored qualitatively. The scores were given as 0 = no expression, 1 = minimal expression, 2 = medium expression, 3 = highest level of expression. A purified control protein with a 6-His tag was used for standardization from blot to blot. The expression experiments were performed at 37°C, 30°C, 20°C, and 12°C for BL21-AI, and 37°C and 30°C for CD43(DE3).

## List of Abbreviations

IPTG: isopropyl-beta-D-thiogalactopyranoside; PCR: polymerase chain reaction; TM: transmembrane; GRAVY: grand average of hydrophobicity; 6-His: 6-histidine affinity tag; CF: cystic fibrosis; TMH: transmembrane helix

## Authors' contributions

VM carried out many of the cloning steps and expression studies, performed some of the calculations pertaining to protein characteristics, and drafted the methods part of the manuscript. FB carried out some of the cloning steps, expression studies, and calculations. CJ conceived of and designed the study, participated in its coordination, selected the proteins for study, performed many of the calculations, assisted in the expression studies, and wrote the manuscript. The final manuscript was approved by all the authors.

## Supplementary Material

Additional file 1 **Table listing the 87 *Pseudomonas aeruginosa *target proteins in the study, including the ORF numbers, gene names, protein names, predicted number of transmembrane helices and percent of amino acids in transmembrane helices, the calculated GRAVY score, and the results of the expression studies at several temperatures in two *E. coli *strains**. Click here for file
