# Supplementary material for: Recombinant Expression Screening of P. aeruginosa Bacterial Inner Membrane Proteins
Source: BMC Biotechnol. 2010 Nov 29;10:83. doi: 10.1186/1472-6750-10-83 (PMC3009615; doi:10.1186/1472-6750-10-83)
Supplement: Additional file 1 — Table listing the 87 Pseudomonas aeruginosa target proteins in the study, including the ORF numbers, gene names, protein names, predicted number of transmembrane helices and percent of amino acids in transmembrane helices, the calculated GRAVY score, and the results of the expression studies at several temperatures in two E. coli strains. [file 1472-6750-10-83-S1.PDF]

Additional File 1. List of selected *Pseudomonas aeruginosa* target proteins

ORF Number	Gene Name	Protein Name	#TM	%Inside	GRAVY	Express	Highest Score	BL21 14°C	BL21 20°C	BL21 30°C	BL21 37°C	C43 30°C	C43 37°C
PA0113	none	probable cytochrome C oxidase assembly factor	8	54	0.807	n	0	0	0	0	0	0	0
PA0205	PotB homologue	probable ABC transporter	6	46	0.752	y	2	2	2	1	0	1	1
PA0255	none	hypothetical	6	57	0.800	y	2	1	2	1	0	0	1
PA0288	speB2	agmatinase	2	13	-0.013	n	0	0	0	0	0	0	0
PA0303	PotH	polyamine ABC transporter	7	51	0857	y	2	0	0	2	0	0	0
PA0325	PotB homologue	probable ABC transporter	6	43	0.618	n	0	0	0	0	0	0	0
PA0341	lgt homologue	prolipoprotein diacylglyceryl transferase	7	53	0.526	y	1	0	1	0	0	0	1
PA0379	ygdD homologue	hypothetical	4	65	1.115	y	1	0	0	1	0	0	0
PA0385	none	hypothetical	3	56	1.059	y	2	0	0	2	0	0	0
PA0397	CzcD homologue	cation efflux system protein	6	39	0.619	y	2	1	2	2	0	0	2
PA0426	mexB	multidrug transporter MexB	13	28	0.274	y	2	0	2	1	1	1	1
PA0435	none	hypothetical	4	17	-0.134	y	2	1	2	1	1	0	1
PA0465	CreD	inner membrane protein CreD	6	26	0.216	y	2	n/a	n/a	n/a	n/a	2	2
PA0563	none	conserved hypothetical protein	3	49	0.426	y	3	0	3	2	1	1	0
PA0661	none	hypothetical	4	61	0.684	y	1	1	0	0	0	0	0
PA0790	none	hypothetical	7	52	0.912	n	0	0	0	0	0	0	0
PA0809	MntH	manganese transporter	10	47	0.825	n	0	0	0	0	0	0	0
PA0918	CybB homologue	bacterial cytochrome b*561*	4	42	0.341	y	3	3	3	3	0	3	3
PA0984	none	colicin immunity protein	3	55	0.721	y	1	0	0	1	0	0	0
PA1279	CobU, cobT homologue	nicotinate-nucleotide dimethylbenzimidazole phosphoribosyl transferase	4	26	0.219	y	3	1	3	2	3	0	1
PA1552	CcoP homologue	probable cytochrome C	2	14	-0.211	y	2	0	0	0	0	1	2
PA1581	sdhC	cytochrome b556	3	47	0.823	y	3	2	1	1	0	3	2
PA1651	BenE homologue	probable transporter	11	60	0.823	y	1	1	1	0	0	0	1
PA1691	unknown	unknown	6	48	1.090	n	0	0	0	0	0	0	0
PA1693	YopR homologue	unknown	4	42	0.672	n	0	0	0	0	0	0	0
PA1773	Cmax	Cmax	2	13	-0.163	y	2	2	1	2	1	1	1
PA1775	none	hypothetical	5	38	0.983	y	3	2	3	1	0	1	2
PA1820	NhaB	sodium/proton antiporter	12	50	0.802	n	0	0	0	0	0	0	0
PA1882	SugE homologue	probable transporter	4	70	1.305	y	1	0	1	0	0	0	0
PA1883	NuoA homologue	probable NADH-ubiquinone/ plastoquinone oxidoreductase	3	47	0.507	y	1	0	1	1	0	1	1
PA1948	rbsC	ribose ABC transporter	7	43	0.940	y	1	0	1	0	0	1	1
PA2059	none	probable permease of	6	34	0.591	n	0	0	0	0	0	0	0

		ABC transporter										
PA2128	cupA1	fimbrial subunit CupA1	3	34	0.421	y	3	3	3	3	3	1	2
PA2340	mtlG homologue	probable maltose/mannitol transporter	7	55	0.727	n	0	0	0	0	0	0	0
PA2397	PvdE	pyoverdine ABC transporter biosynthesis protein	6	27	0.155	y	3	2	3	3	0	0	1
PA2533	none	probable sodium/alanine symporter	10	48	0.809	y	1	0	1	0	0	0	0
PA2541	none	probable CDP-alcohol phosphatidyl transferase	3	28	0.697	y	3	2	3	3	0	1	2
PA2628	none	hypothetical	9	60	0.741	n	0	0	0	0	0	0	0
PA2777	yfdC homologue	hypothetical	6	39	0.251	y	3	0	3	1	0	0	1
PA2811	YodH homologue	probable ABC transporter	7	54	0.893	y	1	0	1	1	0	0	0
PA2830	htpX	heat shock protein and zinc metallopeptidase	4	28	0.417	n	0	0	0	0	0	0	0
PA3039	none	probable transporter	11	51	0.752	n	0	0	0	0	0	0	0
PA3107	MetZ	O-succinylhomoserine sulfhydrylase	2	12	-0.038	y	3	3	3	3	3	1	2
PA3141	WbpM (LpsB homologue)	nuceleotide sugar epimerase/dehydratase	7	23	0.096	n	0	0	0	0	0	0	0
PA3176	glts	sodium/glutamate symporter	12	62	0.857	y	1	0	0	0	0	0	1
PA3189	gltF	probable ABC transporter	6	44	0.585	n	0	0	0	0	0	0	0
PA3210	trkH	potassium uptake protein	13	55	0.743	y	1	0	1	0	0	0	0
PA3217	CyaB	adenylate cyclase	4	19	0.124	y	3	3	3	3	3	2	2
PA3331	none	cytochrome P450	2	10	-0.210	y	3	2	3	3	3	1	3
PA3391	NosR	regulatory protein NosR	8	25	0.074	y	2	1	2	1	0	1	1
PA3395	NosY	NosY	6	45	1.120	y	1	0	0	0	0	0	1
PA3474	YigM homologue	hypothetical	10	70	0.741	n	0	0	0	0	0	0	0
PA3553	ArnC	probable glycosyl transferase	2	15	0.193	y	3	2	3	0	0	2	3
PA3603	dgkA	diacylglycerol kinase	3	45	0.743	y	1	0	1	1	0	0	0
PA3651	CdsA	phosphatidate cytidylyl transferase	7	54	0.897	n	0	0	0	0	0	0	0
PA3690	none	probable metal-transporting P-type ATPase	5	14	0.177	y	3	1	2	3	1	1	1
PA3773	none	hypothetical	12	62	1.042	y	2	0	0	2	0	0	0
PA3890	None	probable ABC transporter	5	52	1.157	n	0	0	0	0	0	0	0
PA3929	CiaB	cyanide insensitive terminal oxidase	8	49	0.863	y	2	0	2	0	0	0	1
PA3936	TauC	probable ABC taurine transporter	5	38	0.800	y	1	0	1	1	0	0	1
PA3955	none	hypothetical	5	53	0.845	n	0	0	0	0	0	0	0
PA4083	CupB4	Chaperone cupB4	2	19	0.003	y	3	1	2	3	2	0	1
PA4153	none	2,3-butanedial dehydrogenase	2	13	0.255	y	3	3	3	3	3	3	3
PA4276	SecE	Secretion protein	3	48	0.990	y	2	0	2	1	0	1	1

PA4417	MurE	UDP-MurNac-tripeptide synthetase	2	8	0.044	y	3	2	2	3	3	2	2
PA4455	yrbE homologue	probable ABC transporter	6	49	0.762	n	0	0	0	0	0	0	0
PA4467	None	hypothetical	8	52	1.037	n	0	0	0	0	0	0	0
PA4479	mrcD	rod shape-determining protein	5	67	0.956	y	1	0	1	1	0	0	1
PA4528	XcpA, PilD	type 4 prepilin peptidase	5	34	0.637	y	2	1	2	1	0	0	1
PA4559	lspA	prolipoprotein signal peptidase	5	64	0.615	y	3	1	3	2	1	1	2
PA4598	MexD	multidrug transporter MexD	14	29	0.401	y	3	0	0	3	2	0	0
PA4614	MscL	mechanosensitive channel	2	31	0.693	y	3	3	3	3	3	3	2
PA4740	Pnp	polyribonucleotide nucleotidyltransferase	3	10	-0.073	y	3	3	3	3	3	0	0
PA4779	none	hypothetical	10	66	1.219	n	0	0	0	0	0	0	0
PA4834	none	hypothetical	9	61	1.163	n	0	n/a	n/a	n/a	n/a	0	0
PA4838	None	hypothetical	8	45	0.665	y	1	0	0	1	0	0	0
PA4990	QacE homologue	SMR multidrug efflux transporter	4	72	1.444	y	2	0	2	1	1	0	0
PA5132	none	hypothetical	7	53	1.031	n	0	0	0	0	0	0	0
PA5168	DctQ homologue	probable dicarboxylate transporter	4	37	0.732	y	2	1	2	1	0	0	0
PA5199	Env2	two-component sensor Env2	2	9	-0.085	y	3	1	2	3	2	2	2
PA5249	None	hypothetical	5	56	0.822	n	0	0	0	0	0	0	0
PA5256	DsbH, DsbB homologue	disulfide bond formation protein	4	50	0.971	y	3	2	3	2	1	0	1
PA5262	AlgZ/FimS	alginate biosynthesis protein	4	21	0.241	y	3	2	3	3	3	3	3
PA5291	BetT homologue	probable choline transporter	12	37	0.419	y	3	0	2	3	2	0	0
PA5361	PhoR	two-component sensor PhoR	2	8	-0.334	y	2	2	1	1	0	2	2
PA5501	ZnuB	ABC zinc transporter	7	65	1.303	n	0	0	0	0	0	0	0
PA5529	YbaL homologue	probable sodium/proton antiporter	16	56	0.989	y	2	0	2	1	0	0	0

Gene name and Protein name were taken from the PseudoCap database. #TM = transmembrane helices predicted by TMPRED, %inside = percentage of amino acids predicted to be inside the transmembrane helices, GRAVY = grand average hydrophobicity, Express = expressed in at least one condition (y = yes, n = no), The Western blots were scored visually, and bands migrating near the predicted molecular weight were scored qualitatively. The scores are given as 0 = no expression, 1 = minimal expression, 2 = medium expression, 3 = highest level of expression. Highest score = highest level of expression in any of the conditions tested.
